# Dependence of crystallographic atomic displacement parameters on temperature (25–150 K) for complexes of horse liver alcohol dehydrogenase

**DOI:** 10.1107/S2059798322008361

**Published:** 2022-09-27

**Authors:** Bryce V. Plapp, Lokesh Gakhar, Ramaswamy Subramanian

**Affiliations:** a Department of Biochemistry and Molecular Biology, The University of Iowa, Iowa City, IA 52252, USA; b Protein and Crystallography Facility, Carver College of Medicine, The University of Iowa, Iowa City, IA 52252, USA; University of Western Australia, Crawley, Australia

**Keywords:** *B* factors, anisotropic displacement parameters, enzyme dynamics, TLS analysis, hydrogen transfer, alcohol dehydrogenases, helium cryostat, X-ray crystallography

## Abstract

Enzymes catalyze reactions by binding and orienting substrates with dynamic interactions. Horse liver alcohol dehydrogenase catalyzes hydrogen transfer with quantum-mechanical tunneling that involves fast motions in the active site. The structures and *B* factors of ternary complexes of the enzyme with NAD^+^ and 2,3,4,5,6-pentafluorobenzyl alcohol or NAD^+^ and 2,2,2-trifluoroethanol were determined to 1.1–1.3 Å resolution below the ‘glassy transition’ in order to extract information about the temperature-dependent harmonic motions, which are reflected in the crystallographic *B* factors. The refinement statistics and structures are essentially the same for each structure at all temperatures. The *B* factors were corrected for a small amount of radiation decay. The overall *B* factors for the complexes are similar (13–16 Å^2^) over the range 25–100 K, but increase somewhat at 150 K. Applying TLS refinement to remove the contribution of pseudo-rigid-body displacements of coenzyme binding and catalytic domains provided residual *B* factors of 7–10 Å^2^ for the overall complexes and of 5–10 Å^2^ for C4N of NAD^+^ and the methylene carbon of the alcohols. These residual *B* factors have a very small dependence on temperature and include local harmonic motions and apparently contributions from other sources. Structures at 100 K show complexes that are poised for hydrogen transfer, which involves atomic displacements of ∼0.3 Å and is compatible with the motions estimated from the residual *B* factors and molecular-dynamics simulations. At 298 K local conformational changes are also involved in catalysis, as enzymes with substitutions of amino acids in the substrate-binding site have similar positions of NAD^+^ and pentafluorobenzyl alcohol and similar residual *B* factors, but differ by tenfold in the rate constants for hydride transfer.

## Abstract 2

The crystallographic temperature factors determined for three different complexes of horse liver alcohol dehydrogenase over the range 25–150 K are relatively independent of temperature and suggest that the *B* factors at 100 K are relevant to catalysis at 300 K.

## 1. Introduction

**Figure 1:**
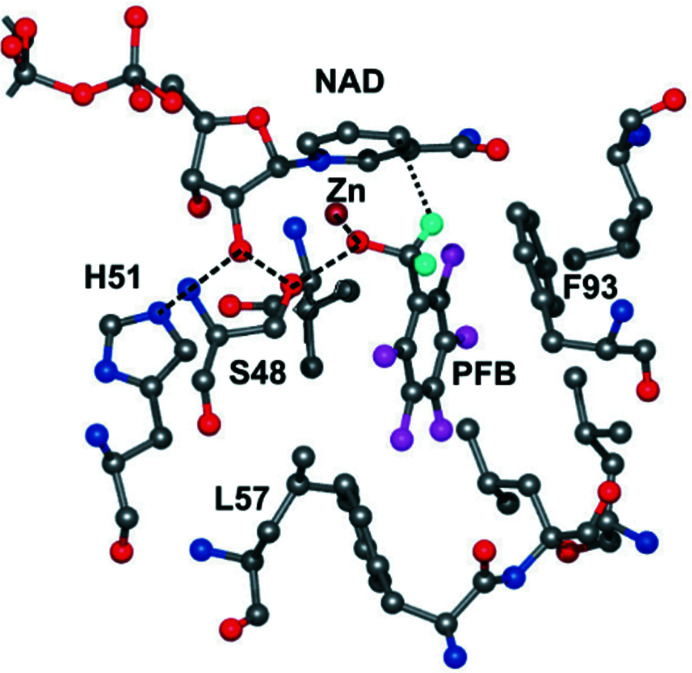
Active site of horse liver alcohol dehydrogenase with NAD^+^ and 2,3,4,5,6-pentafluorobenzyl alcohol (PFB) from the structure determined to 1.14 Å resolution (PDB entry 4dwv; Plapp & Ramaswamy, 2012 ▸). Typical atomic coloring is used, but with cyan for H atoms and magenta for F atoms of PFB, violet for the catalytic Zn atom and gray for the P atom of NAD^+^. A dashed line connects the *pro-R* hydrogen of the alcohol to the *re* face of C4N in the nicotinamide ring. A hydrogen-bond network includes the O atom of PFB, OG of Ser48, ribose O2B of NAD^+^ and NE2 of His51.

X-ray crystallography of enzymes and their complexes provides information about the coordinates of the three-dimensional structures and the motions of protein domains and atoms that are reflected in the atomic displacement parameters, also called ‘temperature’ or *B* factors (Sun *et al.*, 2019 ▸). Atomic resolution structures of alcohol dehydro­genases suggest that hydrogen can be transferred directly from the methylene C atom of the alcohol to C4N of the nicotin­amide ring of the coenzyme NAD^+^ with a ground-state distance of 3.4–3.5 Å (Fig. 1 ▸), and the *B* factors (determined at 100 K) suggest that there would be sufficient motion of the enzyme and substrate atoms in the ternary complexes for hydrogen to be transferred at a distance of 2.6–2.7 Å between the reacting carbons, as estimated from quantum-mechanical calculations (Plapp & Ramaswamy, 2012 ▸; Plapp *et al.*, 2017 ▸; Agarwal *et al.*, 2000 ▸; Alhambra *et al.*, 2000 ▸; Webb *et al.*, 2000 ▸; Villà & Warshel, 2001 ▸; Luo & Bruice, 2001 ▸; Cui *et al.*, 2002 ▸; Roston & Kohen, 2010 ▸; Nagel & Klinman, 2010 ▸).

Because hydrogen has a small mass and a wave-like property, it can tunnel through the classical transition-state energy barrier (Cha *et al.*, 1989 ▸; Nagel & Klinman, 2010 ▸; Klinman & Kohen, 2013 ▸). Kinetic isotope effects and temperature dependencies in the oxidation of benzyl alcohols with protium, deuterium or tritium on the methylene C atom support the existence of hydrogen tunneling in alcohol dehydrogenases (Cha *et al.*, 1989 ▸; Bahnson *et al.*, 1993 ▸; Kohen *et al.*, 1999 ▸; Rubach *et al.*, 2001 ▸; Tsai & Klinman, 2001 ▸). It has been proposed that fast (nanosecond to picosecond) motions of the protein during the reorganization in the enzyme–substrate complex are coupled to the formation of the ‘tunneling-ready state’ for the transfer of hydrogen (Nagel & Klinman, 2010 ▸; Klinman & Kohen, 2013 ▸).

The magnitudes and directions of such harmonic motions might be estimated from crystallographic *B* factors, but these include contributions from ‘static’ disorder due to different positions of rigid-body groups in the unit cells and from thermally activated dynamic ‘disorder’ due to global domain and atomic displacements or motions (Winn *et al.*, 2001 ▸, 2003 ▸). Determination of the temperature dependence of the *B* factors ‘may allow one to separate the dynamic and static contributions’ (Winn *et al.*, 2003 ▸; Helliwell, 2022 ▸).

Previous studies of other proteins have determined *B* factors as a function of temperature and have generally found a ‘glassy transition’ in the range from 110 to 200 K, which involves anharmonic, collective motions of the protein and solvent, but is not fully understood (Teeter *et al.*, 2001 ▸; Ringe & Petsko, 2003 ▸; Kim *et al.*, 2011 ▸; Tournier *et al.*, 2005 ▸). Crystallography of ribonuclease from 98 to 320 K shows that the *B* factors increase with increasing temperature, with a distinct upward change in slope at ∼180–200 K (Tilton *et al.*, 1992 ▸). Crystallography of crambin from 100 to 300 K shows an abrupt increase in slope for the *B* factors at ∼180 K (Teeter *et al.*, 2001 ▸). Crystallography of thaumatin shows a change at ∼110 K (Kim *et al.*, 2011 ▸). Extensive analysis of cyclophilin A shows that the *B* factors are relatively temperature independent between 100 and 280 K, but there is complex conformational heterogeneity as the temperature is varied between 100 and 310 K (Keedy *et al.*, 2015 ▸). Crystallography of lysozyme from 113 to 178 K also shows a change in the dependence at ∼150 K (Joti *et al.*, 2002 ▸). Limited data have been obtained below 100 K, where global motions and rotamer conformations are ‘frozen’ so that only residual (internal or harmonic) atomic displacements would remain (Ringe & Petsko, 2003 ▸; Joti *et al.*, 2002 ▸). X-ray crystallography of myoglobin from 40 to 300 K did not show a glassy transition for the protein, perhaps because solvent channels are lacking in the crystals (Chong *et al.*, 2001 ▸; Joti *et al.*, 2002 ▸). A study of aldose reductase reported an increase in the *B* factor of 1.7 Å^2^ from 15 to 60 K (Petrova *et al.*, 2006 ▸).

Anisotropic displacement parameters include contributions from pseudo-rigid-body displacements of protein domains, which can be analyzed and subtracted from the overall *B* factors by the refinement of TLS (translation/libration/screw rotation) parameters followed by maximum-likelihood refinement in *REFMAC* to provide ‘residual’ *B* factors (Winn *et al.*, 2001 ▸, 2003 ▸). Although TLS refinement for analyzing protein motions may not be definitive (Moore, 2009 ▸), there is a good biochemical rationale for this study. Four TLS groups, corresponding to the two coenzyme-binding domains of the dimer (residues 176–318 and the NAD^+^) and the two catalytic domains (residues 1–175, 319–374 and the two Zn atoms and the alcohol), are appropriate for this enzyme. The catalytic and coenzyme-binding domains are connected by a hinge region that was identified by comparing the structure of the open conformation of the apoenzyme with the closed holoenzyme, where a rotation of about 10° closes the cleft between the domains where the substrates bind (Eklund *et al.*, 1981 ▸; Colonna-Cesari *et al.*, 1986 ▸). Molecular-dynamics studies suggest that anticorrelated motions of the domains may contribute to catalysis (Luo & Bruice, 2004 ▸, 2007 ▸). The ligands bound to the protein are included in the TLS groups so that the residual displacement parameters for the surrogate reacting atoms (C4N of the coenzyme and the methylene C atom of the bound alcohol) can be estimated. The residual *B* factors include information about local harmonic motions that are relevant to the fast (picosecond to femtosecond) dynamics of quantum-mechanical tunneling for hydrogen transfer in the pre-organized enzyme–substrate complexes (Winn *et al.*, 2001 ▸; Bahnson *et al.*, 1993 ▸; Schwartz, 2013 ▸; Cheatum & Kohen, 2013 ▸; Kohen, 2015 ▸). Rate constants for hydrogen transfer have been determined with transient kinetics and simulation for comparison with the *B* factors (Sekhar & Plapp, 1990 ▸; Shearer *et al.*, 1993 ▸; Kim & Plapp, 2020 ▸).

The G173A mutant enzyme was also studied because the substitution in the hinge region could affect the conformational change, even though the steady-state kinetic constants are very similar to those for the wild-type enzyme and the X-ray structure shows that the methyl group of Ala173 is accommodated in a pre-existing cavity (Shanmuganatham *et al.*, 2018 ▸). Moreover, the mounting pins for crystallography were stabilized with expoxy glue, which was suggested to reduce crystal motion in the cryostream.

In the present study, the temperature dependence of *B* factors was determined by X-ray crystallography of three ternary complexes of horse liver alcohol dehydrogenase from 25 to 85 K with a helium cryostat and from 100 to 150 K with a nitrogen cryostat. The structures resemble the expected ground-state structures for catalysis (Figs. 1 ▸ and 2 ▸) and are essentially identical over the temperature range. The unit-cell volumes increase slightly with increasing temperature. There is no evidence of changes in the protonation state of histidine residues, the p*K* values of which should increase significantly at cryogenic temperatures. The overall and residual *B* factors for the protein, C4N of NAD^+^ and the methylene C atom of the alcohol obtained by TLS refinement are almost temperature independent. Nevertheless, the residual *B* factors at 100 K suggest that harmonic motions are sufficient for hydride transfer at 300 K, which is in agreement with molecular-dynamics simulations.

## 2. Methods

### 2.1. Crystallization

Crystals of dimeric, wild-type (natural) horse liver alcohol dehydrogenase 1E (ADH; EC 1.1.1.1; *Equus caballus*; GenBank M64864; UniProt entry P00327; 79 951 Da with four Zn atoms; obtained from Roche Molecular Biochemicals) complexed with NAD^+^ and 2,2,2-trifluoroethanol (TFE) or 2,3,4,5,6-pentafluorobenzyl alcohol (PFB), or of recombinant G173A ADH complexed with NAD^+^ and PFB, were prepared as described previously (Plapp & Ramaswamy, 2012 ▸; Shanmuganatham *et al.*, 2018 ▸). Briefly, 10 mg ml^–1^ enzyme (about 1 ml in 6.5 mm diameter washed dialysis tubing) was dialyzed against 10 ml 50 m*M* ammonium *N*-[tris(hydroxy­methyl)methyl]-2-aminoethanesulfonate, 0.25 m*M* EDTA buffer pH 7.0 at 4°C with 1 m*M* LiNAD^+^ (Roche) and 100 m*M* TFE or 10 m*M* PFB (Aldrich) as the concentration of 2-methyl-2,4-pentanediol (Kodak, treated with activated charcoal) was increased over some days to 12–15%, where crystals formed, and finally increased to 25%, which serves as a cryoprotectant. The well formed block crystals (≥0.2 mm on all sides) were mounted on fiber loops (Hampton Research) and flash-vitrified by plunging them into liquid nitrogen for storage and transport.

### 2.2. X-ray crystallography

Data were collected for these complexes using an ADSC Quantum 315r detector on the 19-ID (SBC) beamline at the Advanced Photon Source (APS) with an X-ray wavelength of 0.9184 Å and a 0.05 × 0.05 mm beam, at a distance of 130 mm, with 3, 4 or 6 s exposures and 0.5° oscillation for ∼760 images covering ∼380° in total, with two passes at φ = 0 and 180°. Data were collected with the helium cryostat (described previously; Petrova *et al.*, 2006 ▸), first at 85 K and then at lower temperatures. After data collection at the lowest temperature, some crystals were stored in liquid nitrogen and later used with the nitrogen cryostat at 100 K and then up to 150 K. For the complex with trifluoroethanol, data were collected with 4 s exposure per frame from the same region of one crystal (∼0.4 × 0.4 × 0.8 mm) at 85, 65 and 45 K (in that order) and then at 25 K after the crystal had been realigned. Some formation of ‘ice’ (O_2_, N_2_) was noted at 25 K. A different region of the same crystal was subsequently used to collect data at 100, 125 and 150 K. For the complex of the wild-type enzyme with PFB, data were collected from one crystal (∼0.2 × 0.2 × 0.2 mm) with 6 s exposure at 75, 50 and 25 K, from a different crystal (for one pass) at 85 K with 6 s exposure and from a third crystal (all from the same batch of crystals) at 100 and 150 K with 4 s exposure. Data for the complex of the G173A enzyme (∼0.2 × 0.2 × 0.6 mm) were collected with 3 s exposures from different regions of one crystal at temperatures at 85 and 50 K. (Data collection was attempted at 15 K, but the temperature fluctuated between 7 and 19 K, perhaps because ice deflected the beam.) Subsequently, data were collected at 120 and 150 K.

Data were scaled and averaged with d*TREK 9.9.9.8L (Pflugrath, 1999 ▸). In general, the overall redundancy was fourfold, with ∼93% completeness overall and 85–93% in the outer shell, with 〈*I*/σ(*I*)〉 ≥ 1 at all temperatures. The data for the complex with TFE at 45 and 65 K have somewhat lower redundancy because the wires to the thermocouple happened to cause shadows on the detector.

The structures were solved by molecular replacement using the coordinates of the ADH–NAD^+^–PFB complex (PDB entry 4dwv) or the complex of ADH with NAD^+^ and TFE (PDB entry 4dxh) as models, which had been revised by adding alternative conformations for about 20 more amino-acid residues per dimer and removing a few water molecules with high *B* factors, generally by applying the criteria described previously (Plapp & Ramaswamy, 2012 ▸). The 2*F*_o_ − *F*_c_ and *F*_o_ − *F*_c_ electron-density maps were examined at levels that would detect atoms (C, N, O) with occupancies greater than about 0.3. Water molecules were added that were connected directly, or via other water molecules, by hydrogen bonds to the protein and had well formed electron density with *B* factors generally less than three times the average *B* factor for the complete model. After refinement with anisotropic displacement parameters, some water molecules developed extreme anisotropy and were deleted. The models for the wild-type enzyme were produced with data to a nominal resolution of 1.1 Å collected at 100 K. The structure of the G173A complex was based on PDB entry 5kj1 with data to 1.2 Å resolution collected at 85 K (Shanmuganatham *et al.*, 2018 ▸).

As listed previously, about 51 of the 748 amino-acid residues in the dimers had alternative conformations (Plapp & Ramaswamy, 2012 ▸). The structural models for all three complexes now have about 70 amino-acid residues per dimer with alternative conformations, and the number of atoms in the protein has increased from 5570 by about 380 atoms to the numbers listed in the tables. Many of these residues are charged and exposed to solvent. Three leucine residues, 57, 116 and 309, are located in the substrate-binding site and have alternative conformations that adapt to the binding of different ligands to the catalytic zinc. The flexible loop, residues 292–299, including proline residues 295 and 296 has slightly different positions in each subunit of the ternary complexes and a different conformation in the apoenzyme (Ramaswamy *et al.*, 1999 ▸). Except for some amino-acid residues that are exposed to solvent, there is good electron density for the amino-acid side chains. The electron-density difference maps show some peaks, probably from methylpentanediol, water or buffer, which are isolated from the protein. The data used to analyze the different temperatures for the wild-type enzyme complexed with PFB were limited to a resolution of 1.2 Å and the data for the G173A enzyme complexed with PFB were limited to 1.3 Å, so that the average 〈*I*/σ(*I*)〉 was >1.0 at 150 K and resolution would not be a variable factor.

The space group for all crystals is *P*1, with one dimeric molecule consisting of 748 amino-acid residues, two NAD^+^ ions, two alcohol molecules and four or five (4*R*)-2-methyl-2,4-pentanediol molecules in the asymmetric unit. The same structure of a complex was used for refinement with the data at all temperatures for that complex. (For consistency, the structures reported previously for PDB entry 4dxh and PDB entry 5kj1 were re-refined with the same versions of the software as used for the rest of the structures.) The structures were refined with riding H atoms by restrained refinement with one round of isotropic and then one round of anisotropic *B* factors with *REFMAC*5.8.0267 for each temperature (Winn *et al.*, 2001 ▸). Models were built and inspected with *O* and were validated with *MolProbity* and *PARVATI* (Jones *et al.*, 1991 ▸; Williams *et al.*, 2018 ▸; Merritt, 1999*b* ▸). The monomer dictionary for NAD^+^ used by *REFMAC* was modified to remove the restraints on planarity and to relax the restraints on bond distances (from 0.02 to 0.10 Å) for the nicotinamide ring. This modified coenzyme is named NAJ in our coordinate files in order to distinguish it from the NAD^+^ (with tight restraints) listed in other structures. The coordinates submitted to the Protein Data Bank are from refinement with anisotropic temperature factors.

### 2.3. TLS refinement

TLS refinement was used to remove contributions from global disorder and to provide residual *B* factors that include information about the local atomic motions of the reacting carbons of the coenzyme and the alcohol. Anisotropic displacement parameters contain four separate contributions, *U* = *U*_crystal_ + *U*_TLS_ + *U*_internal_ + *U*_atom_, where *U*_crystal_ applies to the unit-cell and lattice disorder, *U*_TLS_ represents translations and librations of pseudo-rigid groups within the asymmetric unit, *U*_internal_ includes various collective motions such as torsional rotations and *U*_atom_ represents displacements of individual atoms (Winn *et al.*, 2001 ▸). (Structures deposited in the Protein Data Bank must add the TLS parameters to the residual *B* factors to provide overall anisotropic *B* factors, but the *B* factors were kept separate for the results in the present study.) For this analysis, four groups were included: the two catalytic domains with bound alcohols and the two coenzyme-binding domains with bound coenzyme. Preliminary experiments suggested that refinements with one TLS group for the whole molecule or with more than four groups also decrease the overall *B* factors, but the residual *B* factors from all studies were similar.

One round (ten cycles) of TLS refinement (described by 20 parameters for each of the four domains) with the initial *B* factors set to 20 Å^2^ was followed by one round (ten cycles) of maximum-likelihood refinement with isotropic *B* factors. Validation with *PARVATI* showed no ‘bad joins’ between connecting TLS groups (Zucker *et al.*, 2010 ▸). With a further round of *REFMAC* refinement with TLS parameters and anisotropic displacement parameters, the residual *B* factors for the atoms had magnitudes similar to those from isotropic refinement, but validation with *PARVATI* showed several bad joins and many atoms with extreme anisotropy, precluding a potential analysis of the anisotropy of atoms of interest.

## 3. Results

### 3.1. Refinement of structures

All of the crystals belong to the same space group (*P*1) and have very similar unit-cell parameters, which change little at different temperatures (Table 1 ▸). The unit-cell volumes for the complexes with TFE and PFB increase by about 0.5% as the temperature increases from 25 to 150 K. The complex with PFB determined previously at 277 K has a volume that is 3.4% larger compared with the complex determined at 100 K. This increase is consistent with previous studies that show an ∼2.3% increase per 100 K over the range from 80 to 320 K, which includes the ‘glassy’ transition (Frauenfelder *et al.*, 1987 ▸; Tilton *et al.*, 1992 ▸).

**Figure 2:**
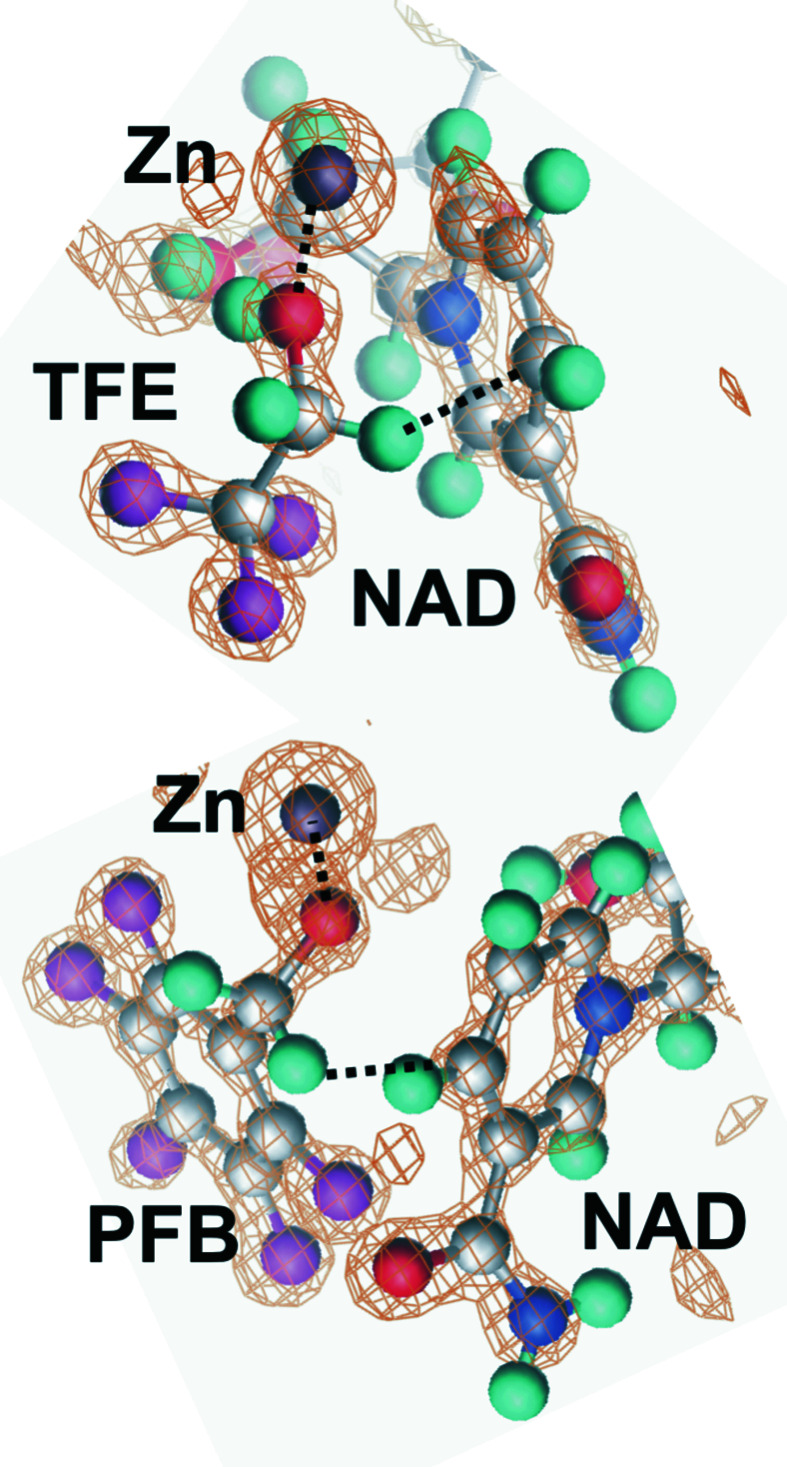
Ligand positions defined by electron densities.

The 17 refined structures of the complexes at various temperatures have good statistics (Tables 2 ▸, 3 ▸ and 4 ▸). The electron-density maps provide atomic resolution, as shown in previous publications (Plapp & Ramaswamy, 2012 ▸; Shanmuganatham *et al.*, 2018 ▸) and clearly define atomic positions for the substrates (Fig. 2 ▸). For each complex, the same model was used for each temperature. As expected, data collected at higher temperatures have a lower 〈*I*/σ(*I*)〉 and somewhat higher *B* factors than at lower temperatures. The overall *B* factors appear to have only a small dependence on the temperature. The overall anisotropy of the protein is typical of many proteins (Merritt, 1999*a* ▸). Subunit *B* of the homodimer generally has higher *B* factors than subunit *A*, apparently due to lattice contacts, as also observed for the *B* factors of C4N of the coenzyme and the methylene C atom (C2 or C7) of the alcohol.

In addition to the use of d*TREK for data processing and *REFMAC* for refinement, we also refined the structures of wild-type ADH with NAD^+^ and PFB with *SHELXL* (Sheldrick, 2015 ▸) and *Phenix* (Liebschner *et al.*, 2019 ▸) and found similar results for the refinement statistics and *B* factors. The magnitudes of the *B* factors differed somewhat because of differences in the programs, but the small temperature dependencies were similar. We also used *XDS* for data processing (but not for scaling) and *REFMAC* for refinements and found similar results.

### 3.2. Correction for radiation decay

X-ray irradiation of a crystal decreases the intensities of the reflections and increases the *B* factors, and corrections are needed so that data at different temperatures can be compared (Garman, 2010 ▸; Kmetko *et al.*, 2006 ▸). Radiation decay is expected to be diminished at lower temperatures and to be cumulative with longer exposures (Hanson *et al.*, 2002 ▸; Meents *et al.*, 2007 ▸, 2010 ▸; Chinte *et al.*, 2007 ▸; Taberman *et al.*, 2019 ▸). Estimates of the decay were obtained by separately processing the images from the first and second passes of each ∼180° rotation at a temperature, providing an approximately twofold redundancy of reflections in each pass and good statistics, *R*_p.i.m._ and 〈*I*/σ(*I*)〉. The data for each pass were used for refinements using the structures determined with the full data set for each temperature, and the *R* values for each partial data set were comparable, whereas the isotropic *B* factors were slightly increased for the second pass compared with the first pass (Tables 5 ▸, 6 ▸ and 7 ▸). The ratio of the *B* factors provides correction factors that are small and not significant beyond the second decimal because of uncertainties in the refinement of *B* factors, differences in data completeness and altered crystal positions, but are accorded extra precision here in order to calculate cumulative corrections. The *B* factors were corrected to represent the state of each crystal before any exposure to X-rays, and the cumulative (multiplicative) correction factor accounts for the previous exposures at other temperatures. Average intensities for reflections from scaling with d*TREK for each crystal increase somewhat as the temperature decreases, but the ratios of intensities for the first and second passes were not as consistent as the ratios of *B* factors. With the helium cryostat (85–25 K) there is slightly less decay than with the nitrogen cryostat at 100 K, as expected.

**Figure 3:**
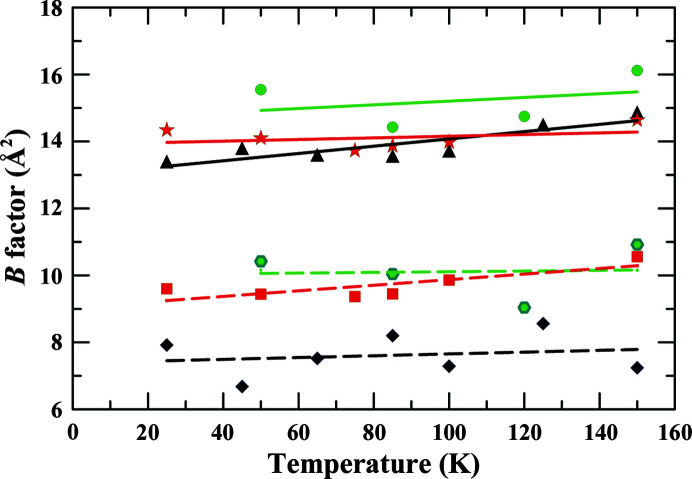
Temperature dependence of overall and residual isotropic *B* factors, corrected for radiation damage. The top three solid lines are overall *B* factors (Tables 5 ▸, 6 ▸ and 7 ▸) for G173A ADH complexed with PFB (green circles), wild-type ADH complexed with PFB (red stars) and wild-type ADH complexed with TFE (black triangles), and the bottom three dashed lines are the residual *B* factors (Table 8 ▸) for G173A ADH (green hexagons), the wild type with PFB (red squares) and the wild type with TFE (black diamonds). The lines are least-squares fits of the data to a straight line. Although the *B* factors show a modest increase with temperature, the fitted values for the slopes have high (>50%) standard errors, except for the overall factors for the complexes with TFE (1.07 ± 0.28 per 100 K) and the residual factors for the wild-type enzyme with PFB (0.83 ± 0.28 per 100 K).

Before or after the small corrections for radiation damage, it appears that the overall isotropic *B* factors are relatively independent of temperature at 100 K and lower (Fig. 3 ▸). The small increases in *B* factors at the lower temperatures may be due to incomplete correction for cumulative radiation damage as the data collection proceeded from higher to lower temperature or to differences in crystal position. Above 100 K there is an apparent increase in *B* factors, which may be an indication of the trend to the glassy transition. The equivalent isotropic *B* factors (calculated from the anisotropic *B* factors) for the atoms of the coenzyme and alcohols show similar temperature dependencies (Tables 2 ▸, 3 ▸ and 4 ▸), but these factors include contributions from the displacements of the crystal lattice and the protein domains to which the ligands are bound. It appears that the anharmonic motions are completely suppressed at 100 K and below, which is consistent with previous studies that show the glassy transition is at higher temperatures.

### 3.3. TLS refinement

TLS analysis and refinement with isotropic *B* factors proceeded with good statistics and led to significantly decreased *B* factors for the overall molecule and for individual atoms of the ligands of interest (Table 8 ▸) compared with the values estimated from refinement with isotropic temperature factors (Tables 5 ▸, 6 ▸ and 7 ▸, Fig. 3 ▸). The residual *B* factors for the complex with TFE are ∼55% of the values with isotropic refinement (see Table 5 ▸), and the residual *B* factors for the complexes with PFB are ∼68% of these values (see Tables 6 ▸ and 7 ▸). It is interesting that the residual *B* factors (Table 8 ▸) are similar to the Wilson *B* factors (Tables 2 ▸, 3 ▸ and 4 ▸). The TLS refinement appears to equalize the overall *B* factors for the *A* and *B* subunits, which shows that the *B* subunit has more ‘mobility’ (larger displacements) than the *A* subunit in the asymmetric unit even though the tertiary structures of the subunits are very similar. Thus, an average *B* factor could be calculated for the atoms of the ligands, C4N of NAD^+^ and the methylene C atom of the alcohols. The major result is that the residual *B* factors have a small temperature dependence. The overall anisotropies of the protein and heteroatoms are substantial, but are relatively independent of temperature, suggesting that the displacements of domains are similar at all temperatures (Winn *et al.*, 2003 ▸; Zucker *et al.*, 2010 ▸).

### 3.4. Relationship of hydride transfer to active-site geometry and residual *B* factors

Substitutions of amino-acid residues (S48T, L57F and F93W) that interact with the substrates in the active site of alcohol dehydrogenase (Fig. 1 ▸) cause significant changes in catalysis, including large changes in catalytic efficiency and a tenfold variation of the rate constant for hydride transfer for the oxidation of benzyl alcohol (Table 9 ▸). The geometries of the coenzyme and alcohol in the active sites of the wild-type and substituted enzymes are very similar and resemble the expected ground-state structures (Plapp & Ramaswamy, 2012 ▸; Kim & Plapp, 2017 ▸, 2020 ▸). The donor–acceptor distances (C4N of NAD^+^ to C7 of benzyl alcohol, from full matrix refinement with *SHELXL*; Sheldrick, 2015 ▸) seem to correlate with the increases in the rate constant for hydride transfer ( 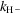 ) for the oxidation of benzyl alcohol determined with transient kinetics and simulation of the full kinetic mechanism. Kinetic isotope effects with the L57F and F93W enzymes show that hydrogen tunneling contributes to catalysis, suggesting a link to protein dynamics (Bahnson *et al.*, 1993 ▸; Tsai & Klinman, 2001 ▸).

Nevertheless, refinement with TLS parameters (as in Section 3.3) shows that the residual *B* factors for the nicotinamide ring and the methylene C atom of the alcohol do not differ significantly among these enzymes (Table 9 ▸). Inspection of the structures suggest that the substitutions of amino-acid residues in the active sites would alter local interactions and conformational changes during the reorganization when the enzyme–NAD^+^–alcohol complex is converted to the enzyme–NADH–aldehyde complex (Kim & Plapp, 2020 ▸; Plapp & Subramanian, 2021 ▸). The residual *B* factors (at 100 K) should not include global conformational fluctuations, but may reflect the local motions that occur during hydride transfer.

## 4. Discussion

### 4.1. Relevance of liver alcohol dehydrogenase for this study

The overall objective of this work is to determine the roles of protein structure and dynamics in enzyme catalysis. Alcohol dehydrogenase is well studied, and it appears that amino-acid residues that are proximal (‘local’; interacting with substrates) in the enzyme–substrate complex contribute especially to catalytic chemistry, whereas distal residues (‘global’; influencing protein stability and fluidity, conformational changes and subunit interactions) contribute to binding and pre-organization of the substrates (Ramaswamy *et al.*, 1999 ▸; Rubach *et al.*, 2001 ▸; Rubach & Plapp, 2003 ▸; Kim & Plapp, 2017 ▸, 2020 ▸; Yahashiri *et al.*, 2014 ▸; Plapp, 2010 ▸; Shanmuganatham *et al.*, 2018 ▸). High-resolution X-ray crystallography provides detailed structures that resemble Michaelis complexes with NAD^+^ and 2,2,2-trifluoroethanol or 2,3,4,5,6-pentafluoro­benzyl alcohol, which are sterically similar to good substrates (Figs. 1 ▸ and 2 ▸) but are not oxidized because the F atoms withdraw electrons from the reaction center and hinder hydride transfer. Ethanol and benzyl alcohol are very good substrates, and benzyl alcohol has been used in studies of the quantum-mechanical hydrogen tunneling (Ramaswamy *et al.*, 1999 ▸; Bahnson *et al.*, 1993 ▸; Rubach *et al.*, 2001 ▸). Enzyme crystallized with NAD^+^ and reactive substrates, such as 4-methylbenzyl or 4-bromobenzyl alcohols, produces abortive complexes with NADH (from reduction of NAD^+^ during the crystallization), and the alcohols bind in an alternative mode (Plapp & Subramanian, 2021 ▸). In contrast, crystallization with fluorinated alcohols inhibits the reduction of NAD^+^ to NADH, and the NAD^+^ and the alcohols in the complexes are positioned appropriately for direct hydride transfer, with the *pro-R* hydrogen of the methylene C atom pointing towards C4N of the nicotinamide ring of the coenzyme at a distance of 3.4–3.5 Å. The nicotinamide rings are slightly puckered (‘strained’), with bond distances and angles that significantly differ from those for either oxidized or reduced coenzyme, as determined by full matrix refinement with *SHELXL* (Kim & Plapp, 2020 ▸; Plapp & Ramaswamy, 2012 ▸). The complexes resemble the potential tunneling-ready state (Roston & Kohen, 2010 ▸). The O atoms of the alcohols are ligated to the catalytic zinc at a distance expected for a zinc alkoxide (1.97 Å) and participate in a low-barrier hydrogen bond (2.52 Å) to the hydroxyl group of Ser48 in the proton-relay system. Structures of complexes with NADH and formamides, corresponding to the products with NADH and aldehydes, provide models for reactive central complexes (Plapp *et al.*, 2017 ▸; Plapp & Subramanian, 2021 ▸).

### 4.2. Temperature effects on the structures

The overall structures of the complexes determined at the different temperatures did not show significant differences. This is expected because flash-vitrification at 77 K in liquid nitrogen freezes the conformations, although the conformations of side chains may change during cooling because the process is not instantaneous, and protons could also move (Joti *et al.*, 2002 ▸; Keedy *et al.*, 2015 ▸; Halle, 2004 ▸; Gakhar & Wiencek, 2005 ▸). Thus, the potential effects of solvents, low temperature and crystallization on the activities and structure of the enzyme should be considered when attempting to relate these cryo-structures to those at ambient temperature.

The overall structures of the complex with pentafluoro­benzyl alcohol determined previously at 2.1 Å resolution at 277 K (PDB entry 1hld; Ramaswamy *et al.*, 1994 ▸) and those reported here determined at 1.1–1.2 Å resolution at 25–150 K are essentially identical, with an r.m.s.d. on α-carbons of ∼0.37 Å. The geometries of the active sites are also almost identical. The earlier structure has 6100 non-H atoms (5570 from protein, 394 waters and 136 heteroatoms) and no modeled alternative conformations of amino-acid side chains, although 11 of the 748 residues showed evidence of multiple conformations in the electron-density map. The present structure has 6981 non-H atoms (5968 from protein, 855 waters and 158 heteroatoms), with alternative conformations for 76 amino-acid residues. Many alternative conformations in the complexes with pentafluorobenzyl alcohol and trifluoro­ethanol have been itemized previously (Plapp & Ramaswamy, 2012 ▸). Some of the amino acids with alternative conformations are in the active site, such as Leu57, Leu116 and Leu309, where they could be directly involved in catalysis. The higher resolution structures determined at cryogenic temperatures help to define the potential dynamics because the alternative conformations represent accessible states.

Nevertheless, the determination of the structures might never be complete because additional alternative conformations could be identified, especially for polar residues on the surface of the molecule. Molecular-dynamics calculations at ambient temperatures based on these structures would expand the range of conformations of amino acids and the motions of domains involved in catalysis. Although it may seem desirable to determine structures at 300 K, the studies with ADH show that the structures determined at cryogenic temperatures are exceptionally informative in showing the conformational states of the enzyme. Above the glassy transition, where the interactions with solvent increase, many more conformations can exist, and anharmonic, external collective motions would be reflected in increased *B* factors (Ringe & Petsko, 2003 ▸).

The protonation states of amino-acid side chains can sometimes be evaluated in structures determined at high resolution (Ahmed *et al.*, 2007 ▸; Howard *et al.*, 2004 ▸; Zhao *et al.*, 2008 ▸). The p*K* values for ionization of the side chains of imidazole, tyrosine and lysine residues are very temperature dependent, and protonation can affect local interactions. The enthalpies of ionization for their side chains are relatively large, and the p*K* values increase as the temperature decreases (Edsall & Wyman, 1958 ▸). For example, the p*K* values for histidine residues in myoglobin determined by NMR increase by ∼0.5 units when the temperature decreases from 322 to 288 K (Bhattacharya & Lecomte, 1997 ▸). The thermodynamic parameters vary for different residues, with an average enthalpy of ionization (Δ*H*°) of 7.2 kcal mol^−1^ and entropy (Δ*S*°) of −6 cal mol^−1^. Histidine hydantoin is a good analog for the imidazole group of a histidine residue in a protein and has a p*K* value of 6.4 at 298 K, with a Δ*H*° of 7.8 kcal mol^−1^ and a Δ*S*° of −3 cal mol^−1^ (Lennette & Plapp, 1979 ▸). Although the heat capacities were not determined in these studies, a simple extrapolation from 298 to 100 K suggests that the p*K* of an imidazole could increase by ∼4 units, resulting in the protonation of both N atoms. The shift in p*K* values of positively charged acids also occurs in organic solvents. The pH* (apparent pH meter reading) for tris(hydroxymethyl)­amino­methane buffer at pH 8 in water at 20°C is 8.0 in 50% 1,2-propanediol:water, but increases to 10.1 at −30°C (Maurel *et al.*, 1975 ▸; Douzou *et al.*, 1976 ▸). Horse liver ADH is stable in cryosolvents, but the apparent p*K* values for binding of NAD^+^ and catalytic activity on ethanol or *p*-nitroso-*N*,*N*-dimethyl­aniline are shifted to higher values in mixed organic/aqueous solvents (Geeves *et al.*, 1983 ▸).

The crystals of alcohol dehydrogenase were prepared at 278 K at pH 7.0, where some histidine imidazole groups could be neutral, and decreasing the temperature could increase the p*K* values and result in protonation. Examination of the structures shows that His67 NE2 is bound to the catalytic zinc, His139 NE2 accepts a hydrogen bond from Arg129 NH, and the other imidazole N atom in each residue donates a hydrogen, indicating that both histidine residues are neutral. Protonation of the second imidazole N atom would certainly cause a structural change, but none was apparent in the structures from 25 to 150 K. Four histidine residues (His34, His105, His138 and His348) might be neutral or protonated, but no changes in structure are apparent. His51 is of special interest in alcohol dehydrogenase because it apparently acts as a base in the proton-relay system to transfer a proton from the alcohol to the bulk solvent (LeBrun *et al.*, 2004 ▸; Plapp & Ramaswamy, 2012 ▸). The position of His51 is also the same in complexes with NADH and a carbonyl compound ligated to the zinc, where a protonated His51 is expected to be a proton donor (PDB entries 5vl0 and 5vn1; Plapp *et al.*, 2017 ▸). Thus, crystallography does not establish the protonation state of His51. Crystallography of the enzyme at 278 K, albeit at lower resolution, shows that the all of the histidine residues also have similar positions (PDB entry 1hld) as at lower temperatures (Ramaswamy *et al.*, 1994 ▸). It appears that the deeply cryo-cooled enzyme has a catalytically active structure.

### 4.3. TLS refinement and temperature dependence of anisotropy and residual *B* factors

TLS refinement provides residual *B* factors that include internal collective motions and local displacements of atoms and that can be related to the average amplitude of motion 〈*x*〉 by *B* = 8π^2^〈*x*〉^2^. Thus, for *B* factors in the range 5–10 Å^2^ the mean displacements 〈*x*〉 could be from 0.25 to 0.35 Å. With a ground-state distance of ∼3.4 Å between C4N of NAD^+^ and the methylene C atom of the alcohol (Plapp & Ramaswamy, 2012 ▸), the equilibrium thermal motions of the atoms could decrease the distance to 2.7–3.2 Å, where hydride transfer would occur, as estimated from various computations (Billeter *et al.*, 2001 ▸; Roston & Kohen, 2010 ▸; Roston *et al.*, 2012 ▸). Of course, the motions will be larger at 298 or 310 K, and anti­correlated motions of the protein domains to which the substrates bind can also contribute to catalysis at higher temperatures (Luo & Bruice, 2004 ▸, 2007 ▸).

The overall and residual *B* factors are relatively independent of temperature and, as expected, do not extrapolate to zero at 0 K (Fig. 3 ▸). Even after the removal of contributions due to global displacements in the crystal lattice and from domain motions, the residual *B* factors probably contain contributions from unidentified sources, such as crystal quality, data collection and processing, refinement protocols, incomplete structural models and radiation damage. TLS refinement should account for rigid-body displacements of domains, but it may not completely account for lattice disorder. Complementary approaches, such as analysis of diffuse scattering, may help to evaluate the applicability of TLS refinement and the contribution of ‘long-range correlated motions across multiple unit cells’ (Moore, 2009 ▸; Meisburger *et al.*, 2020 ▸). The X-ray diffraction images are accessible from SBGRID for further studies, as recommended (Helliwell, 2022 ▸; see Section 5).

The *B* factors for the crystals with PFB were larger than those with TFE, suggesting that the crystal quality is a factor. The mounting stems were stabilized with expoxy glue for the G173A ADH crystals (Alkire *et al.*, 2008 ▸), but this did not seem to affect the mosaicity or the *B* factors as a function of temperature in the different cryostreams.

Previous studies show that crystallographic *B* factors increase with increasing temperature. Ultrahigh-resolution (0.81 Å) crystallography of aldose reductase (using the same equipment as in the present study) showed that the Wilson *B* factor increased from 4.5 Å^2^ at 15 K to 6.2 Å^2^ at 60 K (a slope of 3.7 Å^2^ per 100 K), but radiation damage could have contributed (Petrova *et al.*, 2006 ▸). X-ray crystallography with normal-mode refinement of myoglobin showed ‘total’ mean-square displacements for all non-H atoms (equivalent to overall *B* factors) that increased from 27 Å^2^ at 40 K to 49 Å^2^ at 300 K and ‘internal’ (separated from total displacements with a TLS model) displacements that increased from 9.5 Å^2^ at 40 K to 21 Å^2^ at 300 K (4.4 Å^2^ per 100 K; Chong *et al.*, 2001 ▸). Normal-mode analysis for myoglobin predicted an increase in internal displacements from zero at 0 K with a slope for the equivalent *B* factor of 4.8 Å^2^ per 100 K (Chong *et al.*, 2001 ▸). Normal-mode refinement with X-ray data for lysozyme show that the glassy transition above 150 K is due to the ‘external’ displacements, which are related to the translational and rotational fluctuations, and that the temperature dependence of the internal component of the displacements approximates the theoretical prediction (Joti *et al.*, 2002 ▸). However, the displacements from 113 to 147 K suggest a ‘flattening’ of the total and internal *B* factors at low temperature. Overall crystallographic *B*-factor values for RNase A were also relatively flat below 160 K. In all of these studies the *B* factors below the glassy transition do not extrapolate to zero at 0 K.

Thermally activated harmonic motions should be decreased, but because of quantum effects will not be eliminated, at 0 K. For instance, crystallography of a small molecule at 0.5 Å resolution at 9 K shows very small ellipsoids of 50% probability for non-H atoms, but the H atoms retain some anisotropic displacements and the methyl groups show some evidence of rotational disorder at temperatures below 100 K (Lübben *et al.*, 2014 ▸). For the much larger proteins, experimental approaches and theoretical methods need to be developed further to relate *B* factors to dynamics.

The present study found relatively small temperature dependencies of the overall or residual *B* factors for the complexes with ADH, but does suggest that crystallography at 100 K or lower provides information that is relevant for higher temperatures. With respect to the objective of estimating residual, or intrinsic, atomic displacement parameters that reflect harmonic motions of reacting atoms at 300 K, it appears that residual *B* factors determined here at 100 K for ADH (5–10 Å^2^; Table 8 ▸) are consistent with the suggestion that fast atomic motions of ∼0.3 Å in the ground state could be sufficient to reach a tunneling-ready state for hydrogen transfer (Kim & Plapp, 2020 ▸).

### 4.4. Relevance of X-ray crystallography for understanding enzyme dynamics and catalysis

Some scientists argue that the X-ray structures of crystalline enzymes (for example at 100 K) are static and do not adequately describe the dynamics and the multitude of conformations that could exist at 300 K. In solution, of course, enzymes have considerable conformational flexibility. Crystallization can restrict some of the dynamics, and different conformational states can be observed in various crystal forms. Nevertheless, structures at 100 K represent energetically accessible states that are a basis for understanding enzyme mechanisms. The present study shows that structures of ADH are similar in the range from 25 to 277 K, although the *B* factors and alternative conformations of side chains vary. Overall structures of RNase A, myoglobin, lysozyme and cyclophilin A determined at ∼100 K are similar to those determined in the range from 277 to 320 K even though local conformations and *B* factors change (Tilton *et al.*, 1992 ▸; Chong *et al.*, 2001 ▸; Joti *et al.*, 2002 ▸; Keedy *et al.*, 2015 ▸).

Structures of ternary complexes of ADH determined by crystallography probably represent catalytically active forms. Cryo-enzymology shows that liver ADH is active in organic solvents and at low temperatures. The temperature dependence of activity and kinetic isotope effects for wild-type ADH studied between +3°C and −50°C in 50% methanol:water showed linear Arrhenius plots, and similar studies with F93W ADH suggested that hydrogen is transferred by quantum-mechanical tunneling (Tsai & Klinman, 2001 ▸). Microspectrophotometry (at 23°C) of crystals of ADH prepared by the procedure used in the present studies showed that the enzyme–NAD(H) complexes can react with alcohols or aldehydes to convert the redox state of the coenzyme, as monitored by absorbance changes at 325 nm (Bignetti *et al.*, 1979 ▸). However, the crystal lattice locks the coenzyme in the closed conformation of the enzyme and the reaction is ‘single-turnover’. The substrate-binding site is accessible to solvent and proton transfers. Thin, microcrystals of apoenzyme, with the open conformation, in ammonium sulfate solutions bind NADH ∼1000 times more slowly than dissolved enzyme (Theorell *et al.*, 1966 ▸), probably because the crystal lattice prevents the conformational change that tightens binding (Sekhar & Plapp, 1988 ▸; Kovaleva & Plapp, 2005 ▸). Structural studies with tetrameric yeast alcohol dehydrogenase show that molecules with the open conformation in crystals can bind and release coenzyme (at 278 K) but molecules in the closed conformation do not (Savarimuthu *et al.*, 2014 ▸; Plapp *et al.*, 2016 ▸; Guntupalli *et al.*, 2021 ▸).

After removing the TLS contributions to the *B* factors, the residual *B* factors do not show a significant temperature dependence (Table 8 ▸) and do not correlate with the rates of hydrogen transfer for ADHs with substitutions in the active site (Table 9 ▸). This might indicate that the harmonic motions make a small contribution to the residual *B* factors or that local reorganizations are required for catalysis. The TLS analysis presented here, molecular-dynamics simulations and analysis of isotope effects certainly indicate that ADH has significant global and local dynamics to form ‘near-attack conformations’ or ‘tunneling-ready states’ (Luo & Bruice, 2001 ▸, 2002 ▸, 2007 ▸; Roston & Kohen, 2010 ▸, 2013 ▸; Roston *et al.*, 2012 ▸). Freezing out local conformational changes (anharmonic motions) at temperatures below the glassy transition might stop enzymatic activity, but glutamate dehydrogenase has activity down to 190 K, which is below the dynamical transition at 220 K (Daniel *et al.*, 1998 ▸). After alcohol dehydro­genase forms productive ground-state structures, the harmonic motions in the complexes estimated from residual *B* factors at 100 K (∼0.3 Å) appear to be sufficient to shorten the donor–acceptor distance between the reacting atoms of the coenzyme and the alcohol to accommodate or facilitate hydrogen tunneling. Molecular-dynamics simulations at ∼300 K also provide magnitudes of motions (equilibrium fluctuations, ∼0.3 Å) of amino-acid residues and substrates that are consistent with those estimated by X-ray crystallo­graphy at 100 K (Luo & Bruice, 2001 ▸; Cui *et al.*, 2002 ▸; Agarwal *et al.*, 2000 ▸; Billeter *et al.*, 2001 ▸). Quantum-mechanical computations suggest that equilibrium motions are most relevant for the dynamics of catalysis (Billeter *et al.*, 2001 ▸; Cui *et al.*, 2002 ▸; Villà & Warshel, 2001 ▸; Alhambra *et al.*, 2000 ▸, 2001 ▸). Nevertheless, further computational analysis of recent structures and biochemical data are needed to understand catalysis by alcohol dehydrogenases (Kim & Plapp, 2020 ▸; Plapp & Subramanian, 2021 ▸).

## 5. Deposition of structural data and diffraction images

Structural data and X-ray diffraction images have been deposited in the RCSB PDB and SBGRID: complexes of wild-type enzyme with NAD^+^ and trifluoroethanol at 25 K (PDB entry 7ua6, https://doi.org/10.15785.SBGRID/886), 45 K (PDB entry 7uc9, https://doi.org/10.15785.SBGRID/887), 65 K (PDB entry 7uca, https://doi.org/10.15785.SBGRID/888), 85 K (PDB entry 7ucu, https://doi.org/10.15785.SBGRID/889), 100 K (PDB entry 4dxh, https://doi.org/10.15785.SBGRID/890), 125 K (PDB entry 7ude, https://doi.org/10.15785.SBGRID/891) and 150 K (PDB entry 7udd, https://doi.org/10.15785.SBGRID/892), complexes of wild-type enzyme with NAD^+^ and pentafluorobenzyl alcohol at 25 K (PDB entry 7udr, https://doi.org/10.15785.SBGRID/893), 50 K (PDB entry 7uec, https://doi.org/10.15785.SBGRID/894), 75 K (PDB entry 7uee, https://doi.org/10.15785.SBGRID/895), 85 K (PDB entry 7uef, https://doi.org/10.15785.SBGRID/896), 100 K (PDB entry 7uei, https://doi.org/10.15785.SBGRID/897) and 150 K (PDB entry 7uej, https://doi.org/10.15785.SBGRID/898), and complexes of the G173A enzyme with NAD^+^ and pentafluorobenzyl alcohol at 50 K (PDB entry 7uhv, https://doi.org/10.15785.SBGRID/899), 85 K (PDB entry 5kj1, https://doi.org/10.15785.SBGRID/900), 120 K (PDB entry 7uhw, https://doi.org/10.15785.SBGRID/901) and 150 K (PDB entry 7uhx, https://doi.org/10.15785.SBGRID/902).

## Supplementary Material

PDB reference: alcohol dehydrogenase, complex with NAD and TFE, 25 K, 7ua6

PDB reference: 45 K, 7uc9

PDB reference: 65 K, 7uca

PDB reference: 85 K, 7ucu

PDB reference: 100 K, 4dxh

PDB reference: 125 K, 7ude

PDB reference: 150 K, 7udd

PDB reference: complex with NAD and PFB, 25 K, 7udr

PDB reference: 50 K, 7uec

PDB reference: 75 K, 7uee

PDB reference: 85 K, 7uef

PDB reference: 100 K, 7uei

PDB reference: 150 K, 7uej

PDB reference: G173A alcohol dehydrogenase, complex with NAD and PFB, 50 K, 7uhv

PDB reference: 85 K, 5kj1

PDB reference: 120 K, 7uhw

PDB reference: 150 K, 7uhx

Diffraction images for PDB entry 7ua6.: https://doi.org/10.157845.SBGRID/886

Diffraction images for PDB entry 7ua9.: https://doi.org/10.157845.SBGRID/887

Diffraction images for PDB entry 7uca.: https://doi.org/10.157845.SBGRID/888

Diffraction images for PDB entry 7ucu.: https://doi.org/10.157845.SBGRID/889

Diffraction images for PDB entry 4dxh.: https://doi.org/10.157845.SBGRID/890

Diffraction images for PDB entry 7ude.: https://doi.org/10.157845.SBGRID/891

Diffraction images for PDB entry 7udd.: https://doi.org/10.157845.SBGRID/892

Diffraction images for PDB entry 7udr.: https://doi.org/10.157845.SBGRID/893

Diffraction images for PDB entry 7uec.: https://doi.org/10.157845.SBGRID/894

Diffraction images for PDB entry 7uee.: https://doi.org/10.157845.SBGRID/895

Diffraction images for PDB entry 7uef.: https://doi.org/10.157845.SBGRID/896

Diffraction images for PDB entry 7uei.: https://doi.org/10.157845.SBGRID/897

Diffraction images for PDB entry 7uej.: https://doi.org/10.157845.SBGRID/898

Diffraction images for PDB entry 7uhv.: https://doi.org/10.157845.SBGRID/899

Diffraction images for PDB entry 5kj1.: https://doi.org/10.157845.SBGRID/900

Diffraction images for PDB entry 7uhw.: https://doi.org/10.157845.SBGRID/901

Diffraction images for PDB entry 7uhx.: https://doi.org/10.157845.SBGRID/902

## Figures and Tables

**Table 1:** Unit-cell parameters of crystals of complexes of alcohol dehydrogenase at various temperatures

Crystal, temperature (K)	*a* (Å)	*b* (Å)	*c* (Å)	α (°)	β (°)	γ (°)	Mosaicity (°)
TFE6, 25	44.17	51.13	92.45	91.88	103.08	109.87	0.63
TFE6, 45	44.19	51.12	92.46	91.90	103.03	109.80	0.65
TFE6, 65	44.19	51.12	92.46	91.89	103.04	109.82	0.63
TFE6, 85	44.19	51.14	92.49	91.90	103.03	109.83	0.61
TFE6, 100	44.25	51.16	92.53	91.90	103.02	109.90	0.65
TFE6, 125	44.28	51.18	92.59	91.90	103.02	109.89	0.64
TFE6, 150	44.25	51.14	92.70	91.92	103.06	109.64	0.59
PFB12, 25	44.42	51.21	92.41	91.72	103.12	110.22	0.73
PFB12, 50	44.41	51.22	92.42	91.72	103.11	110.22	0.72
PFB12, 75	44.40	51.23	92.43	91.72	103.11	110.21	0.69
PFB15, 85	44.28	51.27	92.50	91.81	103.05	110.05	0.48
PFB16, 100	44.31	51.27	92.52	91.90	103.01	110.12	0.66
PFB16, 150	44.38	51.33	92.63	91.86	103.05	110.14	0.71
1hld, 277†	44.50	52.30	93.15	93.12	102.82	109.40	
G173A, 50	44.30	51.48	92.32	91.89	103.04	110.16	0.66
G173A, 85	44.31	51.50	92.42	91.82	103.05	110.12	0.62
G173A, 120	44.37	51.55	92.57	91.80	103.02	110.19	0.57
G173A, 150	44.39	51.56	92.71	91.79	103.04	110.10	0.55

† PDB entry 1hld with PFB solved at 2.4 Å resolution (Ramaswamy *et al.*, 1994 ▸).

**Table 2:** Data-collection and refinement statistics for wild-type ADH complexed with NAD^+^ and 2,2,2-trifluoroethanol Data were refined in the resolution range 20–1.1 Å. Values in parentheses are for the outer shell (1.14–1.10 Å).

Temperature (K)	25	45	65	85	100	125	150
PDB code	7ua6	7uc9	7uca	7ucu	4dxh	7ude	7udd
No. of reflections							
Total	1142316	1065509	994485	1138600	1131238	1136081	1136366
Unique	278830	277899	277772	279168	277733	278692	278542
Completeness (%)	94.2 (90.8)	93.8 (89.9)	93.8 (89.8)	94.2 (90.7)	93.6 (88.9)	93.7 (88.9)	93.5 (88.5)
Multiplicity	4.06 (4.09)	3.78 (3.78)	3.54 (3.56)	4.04 (4.03)	4.01 (4.01)	4.00 (4.01)	4.01 (4.01)
*R*_p.i.m._ (%)	3.5 (23.0)	3.0 (24.5)	3.2 (25.4)	3.4 (24.2)	2.8 (23.8)	2.9 (30.7)	3.2 (32.1)
〈*I*/σ(*I*)〉	9.1 (2.8)	9.8 (2.2)	9.5 (2.3)	9.0 (2.5)	10.0 (2.0)	8.9 (1.3)	8.2 (1.1)
*R*_work_, *R*_free_ (%)	12.2, 14.0	12.6, 15.2	12.6, 14.6	12.4, 14.2	12.8, 15.0	13.4, 16.0	13.5, 16.0
*R*_free_ reflections	2775	2760	2757	2768	2751	2759	2762
R.m.s.d., bond lengths (Å)	0.013	0.013	0.013	0.013	0.013	0.014	0.015
R.m.s.d., angles (°)	1.88	1.93	1.91	1.88	1.92	1.95	1.98
E.s.u.† (Å)	0.017	0.019	0.019	0.018	0.018	0.021	0.022
*MolProbity* scores‡	0.89, 98th; 0.96, 98th	1.21, 98th; 1.03, 98th	0.97, 98th; 1.01, 98th	0.81, 98th; 0.91, 99th	0.56, 98th; 0.89, 99th	0.81, 98th; 0.91, 99th	0.97, 98th; 1.01, 98th
Ramachandran§ (%)	97.04	97.04	97.85	97.31	96.91	97.18	97.04
Wilson *B* (Å^2^)	9.0 ± 0.2	9.5 ± 0.3	9.4 ± 0.2	9.3 ± 0.2	9.3 ± 0.3	10.0 ± 0.3	10.4 ± 0.4
*REFMAC B* ¶ (Å^2^)	13.8	14.6	14.3	14.1	14.3	15.4	16.4
*B*, protein atoms (Å^2^)	13.6	14.4	14.2	13.9	14.1	15.2	16.2
*B*, waters (Å^2^)	24.9	26.1	25.5	25.0	25.4	27.3	28.3
*B*, heteroatoms (Å^2^)	16.7	17.6	17.2	17.0	17.2	18.3	19.5
Protein anisotropy	0.54	0.54	0.55	0.55	0.55	0.55	0.55
*B*, C4N (*A*, *B*)†† (Å^2^)	8.0, 9.6	8.7, 10.4	8.4, 10.7	8.5, 10.3	8.5, 10.5	9.3, 11.8	10.1, 12.3
*B*, C2 (*A*, *B*)†† (Å^2^)	9.6, 11.6	10.0, 12.7	9.8, 13.1	9.6, 12.4	10.2, 13.0	11.9, 14.5	12.5, 15.2

† Estimated standard uncertainty for coordinates based on maximum likelihood.

‡ *MolProbity* clashscore, percentile; score, percentile.

§ Ramachandran angles in favored region; there were no outliers.

¶ Anisotropic *B* factors for 6983 total non-H atoms and analysis with the *PARVATI* server for 5935 protein atoms, 904 water molecules and 144 heteroatoms: four zinc ions, two TFE molecules, two NAD^+^ ions and four MRD molecules.

†† Anisotropic *B* factors for atoms of NAD^+^ and TFE in subunits *A* and *B*.

**Table 3:** Data-collection and refinement statistics for wild-type ADH complexed with NAD^+^ and 2,3,4,5,6-pentafluorobenzyl alcohol Data were refined in the resolution range 20–1.2 Å. Values in parentheses are for the outer shell (1.24–1.20 Å).

Crystal No., temperature (K)	12, 25	12, 50	12, 75	15, 85	16, 100	16, 150
PDB code	7udr	7uec	7uee	7uef	7uei	7uej
Reflections						
Total	844478	846271	848920	446447	891128	895111
Unique	213289	212422	213383	215003	215903	216583
Completeness (%)	93.2 (87.6)	92.8 (86.2)	93.2 (87.1)	94.4 (91.3)	93.9 (88.0)	94.2 (89.3)
Multiplicity	3.91 (3.90)	3.92 (3.90)	3.92 (3.88)	2.04 (2.05)	4.05 (4.07)	4.05 (4.09)
*R*_p.i.m._ (%)	2.7 (23.9)	2.6 (22.8)	2.6 (19.4)	3.8 (29.2)	3.0 (29.8)	3.3 (32.3)
〈*I*/σ(*I*)〉	10.7 (2.1)	10.7 (2.0)	11.6 (2.6)	8.9 (2.0)	8.8 (1.0)	7.9 (1.0)
*R*_work_, *R*_free_ (%)	12.4, 15.9	12.5, 16.1	12.2, 14.4	12.4, 14.8	12.7, 15.0	12.9, 15.4
*R*_free_ reflections	2131	2115	2158	3198	2141	2159
R.m.s.d., bond lengths (Å)	0.016	0.016	0.015	0.015	0.016	0.017
R.m.s.d., angles (Å)	2.08	2.05	2.02	2.01	2.04	2.04
E.s.u.† (Å)	0.025	0.025	0.023	0.025	0.025	0.026
*MolProbity* scores‡	0.80, 99th; 0.91, 99th	0.96, 99th; 0.99, 99th	1.20, 98th; 1.02, 98th	1.28, 98th; 1.03, 99th	1.44, 98th; 1.05, 98th	1.12, 98th; 1.01, 99th
Ramachandran§ (%)						
Favored	97.24	96.91	97.04	97.18	97.04	96.91
Outliers	0	0	0	0	0	0.13
Wilson *B* factor (Å^2^)	10.8 ± 0.4	10.4 ± 0.4	9.9 ± 0.3	9.5 ± 0.6	10.6 ± 0.3	11.4 ± 0.4
*REFMAC B* [Table-fn tfn10] (Å^2^)	16.8	16.1	15.5	14.8	15.7	16.8
*B*, protein atoms (Å^2^)	16.6	16.0	15.4	14.6	15.5	16.6
*B*, heteroatoms (Å^2^)	19.6	18.3	17.5	17.0	18.0	19.3
*B*, waters (Å^2^)	29.5	28.4	27.2	26.7	28.0	29.6
Protein anisotropy	0.53	0.53	0.53	0.53	0.54	0.54
*B*, C4N (*A*, *B*)[Table-fn tfn11] (Å^2^)	12.0, 13.7	11.2, 12.6	9.8, 11.4	8.86, 11.13	10.9, 12.6	10.3, 14.5
*B*, C7 (*A*, *B*)[Table-fn tfn11] (Å^2^)	12.9, 15.1	12.1, 13.6	10.0, 12.1	9.64, 11.45	12.6, 14.5	14.4, 17.1

† Estimated standard uncertainty for coordinates based on maximum likelihood.

‡ *MolProbity* clashscore, percentile; score, percentile.

§ The only outlier is *B*368 Ile at 150 K.

¶ Anisotropic *B* factors for 6981 total non-H atoms and analysis with the *PARVATI* server: heteroatoms are four zinc ions, two PFB molecules, four NAD^+^ ions and four MRD molecules.

†† Anisotropic *B* factors for atoms of NAD^+^ and PFB in subunits *A* and *B*.

**Table 4:** Data-collection and refinement statistics for G173A ADH complexed with NAD^+^ and 2,3,4,5,6-pentafluorobenzyl alcohol Data were refined in the resolution range 20–1.3 Å. Values in parentheses are for the outer shell (1.35–1.30 Å).

Temperature (K)	50	85	120	150
PDB entry	7uhv	5kj1	7uhw	7uhx
Reflections				
Total	688692	692927	704901	705815
Unique	169833	171761	169475	171184
Completenes (%)	94.6 (92.9)	95.5 (92.8)	93.9 (93.5)	94.5 (89.4)
Multiplicity	3.95 (3.86)	3.97 (3.89)	4.01 (3.97)	4.01 (3.95)
*R*_p.i.m._ (%)	0.031 (0.23)	0.030 (0.17)	0.029 (0.22)	0.034 (0.29)
〈*I*/σ(*I*)〉	9.5 (1.6)	11.4 (3.4)	10.0 (1.7)	8.8 (1.2)
*R*_work_, *R*_free_ (%)	13.0, 16.7	12.0, 15.0	12.9, 16.5	13.1, 16.6
*R*_free_ reflections	2564	2590	2538	2574
R.m.s.d., bond lengths (Å)	0.017	0.017	0.017	0.017
R.m.s.d., angles (°)	2.15	2.18	2.16	2.14
E.s.u.†	0.034	0.027	0.032	0.034
*MolProbity* scores‡	1.20, 99th; 1.01, 99th	1.04, 99th; 0.96, 99th	1.04, 99th; 0.98, 99th	1.52, 99th; 1.05, 99th
Ramachandran§	97.18	97.18	97.04	97.18
Wilson *B* factor (Å^2^)	10.3 ± 0.4	10.2 ± 0.3	10.8 ± 0.4	12.5 ± 0.4
*REFMAC B* ¶ (Å^2^)	16.3	15.4	16.3	17.8
*B*, protein atoms (Å^2^)	16.3	15.3	16.2	17.7
*B*, waters (Å^2^)	27.9	26.7	28.2	30.7
*B*, heteroatoms (Å^2^)	12.7	16.9	20.0	20.3
Protein anisotropy	0.52	0.55	0.52	0.49
*B*, C4N (*A*, *B*)†† (Å^2^)	9.1, 12.4	8.5, 11.9	9.7, 13.1	11.3, 18.8
*B*, C7 (*A*, *B*)†† (Å^2^)	12.4, 13.4	10.6, 13.1	13.3, 16.0	16.3, 21.4

† Estimated standard uncertainty for coordinates based on maximum likelihood.

‡ *MolProbity* clashscore, percentile; score, percentile.

§ Ramachandran angles in favored region (%); there were no outliers.

¶ Anisotropic *B* factors for a total of 6991 non-H atoms and analysis with the *PARVATI* server: heteroatoms are four zinc ions, two PFB moelcules, two NAD^+^ ions and four MRD molecules.

†† Anisotropic *B* factors for atoms of NAD^+^ and PFB in subunits *A* and *B*.

**Table 5:** Correction for X-ray damage for wild-type ADH complexed with NAD^+^ and TFE Cumulative correction was used for data at 85 → 65 → 45 K and 100 → 125 → 150 K, and a simple correction for 85, 25 and 100 K, based on a smoothed graph of the ratio of *B* factors (second/first passes) versus temperature (new regions of the crystal for 85, 25 and 100 K). The corrected *B* factor was calculated by dividing the *B* factor from refinement by the cumulative correction factor.

Temperature (K), pass	Wilson *B* ± SE (Å^2^)	*REFMAC R*, *R*_free_, isotropic (%)	*REFMAC B*, isotropic (Å^2^)	Observed second/first-pass *B*	Cumulative correction factor	Corrected *B*, isotropic (Å^2^)
25	8.98 ± 0.21	16.2, 17.7	13.46	1.0044	1.0044	13.40
25, 1st	8.66 ± 0.40	16.6, 18.3	13.50			
25, 2nd	8.85 ± 0.40	16.6, 18.4	13.56			
45	9.46 ± 0.28	16.5, 18.5	14.21	1.0056	1.023	13.81
45, 1st	9.26 ± 0.39	16.7, 18.8	14.14			
45, 2nd	9.81 ± 0.11	16.8, 18.7	14.22			
65	9.33 ± 0.28	16.5, 18.1	13.91	0.998	1.016	13.60
65, 1st	9.15 ± 0.40	16.7, 18.7	13.92			
65, 2nd	9.68 ± 0.10	17.2, 18.4	13.89			
85	9.30 ± 0.22	16.3. 17.8	13.61	1.018	1.018	13.37
85, 1st	9.00 ± 0.40	16.7, 18.3	13.57			
85, 2nd	9.11 ± 0.40	17.0, 18.9	13.82			
100	9.33 ± 0.25	16.7, 18.3	13.88	1.0058	1.012	13.72
100, 1st	8.96 ± 0.40	16.9, 18.9	13.79			
100, 2nd	9.12 ± 0.42	16.9, 18.4	13.87			
125	9.99 ± 0.31	17.1, 18.7	14.95	1.013	1.030	14.51
125, 1st	9.82 ± 0.50	17.0, 19.2	14.98			
125, 2nd	9.92 ± 0.51	17.4, 19.2	15.17			
150	10.35 ± 0.36	17.1, 19.5	15.78	1.041	1.061	14.87
150, 1st	9.88 ± 0.49	17.3, 19.4	15.33			
150, 2nd	10.16 ± 0.64	17.6, 19.6	15.96			

**Table 6:** Correction for X-ray damage for wild-type ADH complexed with NAD^+^ and PFB Exposure was 6 s per image at 25, 50 and 75 K of the same region of the crystal; 6 s at 85 K and 4 s at 100 and 150 K on adjacent regions.

Crystal No., temperature (K), pass	Wilson *B* ± SE (Å^2^)	*REFMAC R*, *R*_free_, isotropic (%)	*REFMAC B*, isotropic (Å^2^)	Observed second/first-pass *B*	Cumulative correction factor	Corrected *B*, isotropic (Å^2^)
12, 25	10.84 ± 0.38	15.7, 17.9	15.34	1.017	1.07	14.34
12, 25, 1st	10.44 ± 0.64	15.8, 17.5	15.13			
12, 25, 2nd	11.01 ± 0.60	16.0, 18.0	15.39			
12, 50	10.43 ± 0.39	15.7, 18.0	14.81	1.012	1.05	14.10
12, 50, 1st	10.07 ± 0.64	16.0, 17.9	14.59			
12, 50, 2nd	10.43 ± 0.60	15.7, 17.9	14.76			
12, 75	9.86 ± 0.33	15.6, 16.4	14.15	1.028	1.03	13.73
12, 75, 1st	9.40 ± 0.63	15.5, 16.4	13.90			
12, 75, 2nd	9.96 ± 0.59	15.7, 16.8	14.29			
15, 85, 1st	9.50 ± 0.58	15.9, 16.9	14.15	1.014	1.02	13.87
16, 100	10.61 ± 0.34	16.3, 17.6	14.62	1.046	1.046	13.98
16, 100, 1st	10.23 ± 0.50	16.3, 17.4	14.18			
16, 100, 2nd	10.88 ± 0.58	16.7, 18.1	14.83			
16, 150	11.44 ± 0.38	16.5, 17.6	15.53	1.061	1.061	14.64
16, 150, 1st	10.83 ± 0.53	16.5, 17.7	14.96			
16, 150, 2nd	12.01 ± 0.60	16.7, 17.8	15.87			

**Table 7:** Correction for X-ray damage for G173A ADH complexed with NAD^+^ and PFB The crystal was realigned for each temperature. Epoxied mounting stems.

Temperature (K), pass	Wilson *B* ± SE (Å^2^)	*REFMAC R*, *R*_free_, isotropic (%)	*REFMAC B*, isotropic (Å^2^)	Observed second/first-pass *B* = correction factor	Corrected *B*, isotropic (Å^2^)
50	10.34 ± 0.39	16.5, 18.0	15.55	1.00	15.55
50, 1st	10.50 ± 0.61	16.5, 18.3	15.24		
50, 2nd	10.71 ± 0.73	16.8, 19.1	15.21		
85	10.15 ± 0.28	15.5, 17.1	14.72	1.02	14.43
85, 1st	9.86 ± 0.41	15.8, 17.7	14.50		
85, 2nd	10.31 ± 0.47	16.1, 17.8	14.79		
120	10.75 ± 0.36	16.5, 18.0	15.41	1.045	14.75
120, 1st	10.77 ± 0.59	16.3, 17.8	14.79		
120, 2nd	11.18 ± 0.60	16.2, 17.7	15.45		
150	12.53 ± 0.45	16.6, 18.1	17.08	1.059	16.11
150, 1st	12.23 ± 0.62	16.7, 19.0	16.33		
150, 2nd	13.05 ± 0.72	17.1, 19.1	17.30		

**Table 8:** Residual *B* factors after TLS refinement of ADH complexes with NAD^+^ and fluoroalcohols The *R*_work_ and *R*_free_, overall residual *B* factor, e.s.u for coordinates (Å), average residual *B* factors for C4N of NAD^+^ and CH_2_ of the fluoroalcohols and the range of these two values, and the overall anisotropy of the protein atoms and the heteroatoms that were included in the TLS groups are given. The TLS contribution is not included in these *B* factors, which are corrected for a little radiation damage with the correction factors in Tables 5–7.

						Anisotropy	
Complex, K	*R*, *R*_free_ (%)	*B* (Å^2^)	E.s.u. (Å)	C4N *B* (Å^2^)	CH_2_ *B* (Å^2^)	Protein atoms	Heteroatoms
WT–TFE					C2	5935 atoms	104 atoms
25	14.5, 15.7	7.92	0.020	5.1 ± 0.4	7.0 ± 0.6	0.45 ± 0.11	0.51 ± 0.11
45	15.2, 17.3	6.68	0.022	3.6 ± 0.4	5.6 ± 0.4	0.37 ± 0.11	0.40 ± 0.13
65	15.1, 16.7	7.52	0.022	4.7 ± 0.2	6.8 ± 0.3	0.41 ± 0.11	0.46 ± 0.14
85	14.7, 16.2	8.20	0.020	5.8 ± 0.4	7.6 ± 0.3	0.47 ± 0.11	0.54 ± 0.13
100	15.2, 16.5	7.29	0.023	4.4 ± 0.4	7.1 ± 0.4	0.41 ± 0.11	0.41 ± 0.11
125	16.2, 18.0	8.56	0.029	5.6 ± 0.3	8.6 ± 0.4	0.46 ± 0.10	0.50 ± 0.13
150	16.0, 18.1	7.24	0.026	4.2 ± 0.4	7.4 ± 0.8	0.42 ± 0.10	0.44 ± 0.13
WT–PFB					C7	5968 atoms	118 atoms
25	15.3, 17.5	9.60	0.030	8.6	8.9	0.45 ± 0.11	0.47 ± 0.10
50	15.0, 17.2	9.44	0.027	8.0	8.6 ± 0.4	0.45 ± 0.11	0.48 ± 0.10
75	14.8, 15.5	9.37	0.026	6.8 ± 0.1	7.9 ± 0.1	0.46 ± 0.11	0.50 ± 0.10
85	14.9, 15.8	9.45	0.027	7.5	8.1 ± 0.2	0.50 ± 0.12	0.56 ± 0.12
100	15.3, 16.1	9.86	0.027	8.1 ± 0.1	10.2 ± 0.2	0.52 ± 0.13	0.62 ± 0.13
150	15.5, 16.4	10.56	0.028	8.8 ± 0.4	12.0 ± 0.1	0.54 ± 0.12	0.63 ± 0.13
G173A–PFB					C7	5959 atoms	118 atoms
50	16.0, 17.4	10.42	0.040	7.8 ± 0.6	8.9 ± 0.4	0.50 ± 0.11	0.56 ± 0.12
85	14.8, 16.4	10.04	0.032	7.4 ± 0.6	8.6	0.52 ± 0.12	0.58 ± 0.11
120	15.8, 17.2	9.04	0.034	6.9 ± 0.3	9.0 ± 0.2	0.45 ± 0.11	0.49 ± 0.12
150	16.3, 17.9	10.92	0.042	9.8 ± 0.9	13.1 ± 0.1	0.51 ± 0.11	0.58 ± 0.12

**Table 9:** Residual *B* factors, rate constants for hydride transfer and geometry of alcohol dehydrogenases with substitutions in the active site complexed with NAD^+^ and pentafluorobenzyl alcohol All structures were refined with data from 20.0 to 1.1 Å resolution collected at 100 K. The rate constant for hydride transfer ( 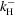 ), the average distance (in the two subunits) between C4N of NAD^+^ and C7 of the alcohol (C4N–C7), the residual isotropic *B* factors for the overall structure (*B*_ave_) and the average and range for the ligands are given.

Mutant	PDB code	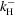 (s^–1^)	C4N–C7 (Å)	*R*, *R*_free_ (%)	E.s.u. (Å)	*B*_ave_ (Å^2^)	C4N *B* (Å^2^)	C7 *B* (Å^2^)
S48T	5kcp	5.4	3.28 ± 0.02	15.5, 17.2	0.024	11.0	9.4 ± 0.5	11.2 ± 0.5
F93W	6owm	36	3.34 ± 0.02	15.9, 16.7	0.022	10.7	8.2 ± 0.6	10.2
WT	4dwv	38	3.36 ± 0.02	15.3, 16.9	0.020	11.5	9.7	10.1 ± 0.3
L57F	6o91	61	3.50 ± 0.03	14.6, 15.1	0.022	10.8	9.5 ± 0.5	11.4 ± 0.5
